# Evaluating the Effectiveness of Jigsaw-Based Learning in Medical Education: Students’ Perceptions and Feedback

**DOI:** 10.7759/cureus.77203

**Published:** 2025-01-09

**Authors:** Archana Nimesh, Gitanjali Goyal, Ramnika Aggarwal

**Affiliations:** 1 Department of Biochemistry, All India Institute of Medical Sciences, Bathinda, Bathinda, IND; 2 Department of Community and Family Medicine, All India Institute of Medical Sciences, Bathinda, Bathinda, IND

**Keywords:** biochemistry, feedback, jigsaw, medical education, perceptions, retention, revision, self-directed learning, students

## Abstract

Background

The medical education system periodically revises the teaching-learning strategies. Medical students find it difficult to cope with pre-clinical subjects due to limited patient exposure and traditional didactic lectures. This study introduced a jigsaw method for revising biochemistry topics and assessed first-year medical students' perceptions and feedback on its effectiveness and implementation in medical education.

Materials and methods

This cross-sectional study enrolled 80 students for a jigsaw exercise on the topic "carbohydrate chemistry." Students were divided into four groups guided by a moderator. Further subgroups were created in each group, and each student was assigned a subtopic in "carbohydrate chemistry." Students with common subtopics were regrouped to self-study the provided study material. Students then reassembled in their original subgroups to teach their respective subtopics to their peers. Thus, it enables all students of a subgroup to learn all subtopics of the main topic in a short time as an interactive team. In the end, students filled out a feedback form providing their opinion about the effectiveness of the exercise.

Results

Out of 80 students, 71 responded to the survey, yielding an 88.75% response rate. Most students opined that the jigsaw exercise enhanced their understanding (N = 59, 83.1%), clarified concepts (N = 54, 76.1%), improved retention (N = 55, 77.5%), and communication skills (N = 59, 83.1%) and that it is a good method to revise topics (N = 58, 81.7%). Most supported its inclusion in the medical curriculum (N = 56, 78.9%) and recommended frequent use (N = 49, 69%).

Conclusion

The jigsaw exercise seems promising for improving students’ understanding, clearing doubts, enhancing retention and communication skills, and fast revision. The authors recommend its inclusion in the curriculum to facilitate self-directed active learning.

## Introduction

The scope of medical education has been consistently evolving, passing through phases like periodic review of the pre-existing teaching-learning strategies and development of new ones with a vision to make the system of medical education better [1], wherein the medical graduates would imbibe the necessary knowledge efficiently to serve the society in the best possible way. However, one of the main challenges medical undergraduates encounter is during their first professional year of studying pre-clinical basic science subjects, wherein their exposure to patients is very limited. Thus, a deeper understanding of the concepts could be difficult when the information is imparted through didactic lectures [2]. Therefore, time and again, newer teaching-learning methods have been introduced in the medical education system to make the learning process more effective, such as flipped classrooms, problem-based discussion, virtual patients, human patient simulations, podcasts, webinars, and other innovative digital tools like computer-aided instruction [3,4]. In our study, we introduced jigsaw as a revision exercise in biochemistry to reinforce the concepts. We have expressed students' perceptions about the effectiveness of the jigsaw activity and their feedback regarding its implementation in medical education.

A jigsaw activity is a cooperative learning [5,6] group activity that enables students to learn a topic actively in a structured manner, covering various aspects of the topic. A jigsaw exercise allows each group student to learn one subpart of a main topic in a self-directed way and teach the same to other students in the group. Thus, all students in that group comprehensively learn the topic from all relevant perspectives. So far, there is just one study conducted on the biochemistry subject by Uppal et al. [7], which has evaluated the feedback of medical students regarding the jigsaw method. The study reported that students found the jigsaw technique effective, but more studies are required to validate the effectiveness of the jigsaw activity. Thus, our study's objective was to analyze students' opinions about the jigsaw activity and make recommendations if found useful.

## Materials and methods

**Figure 1:**
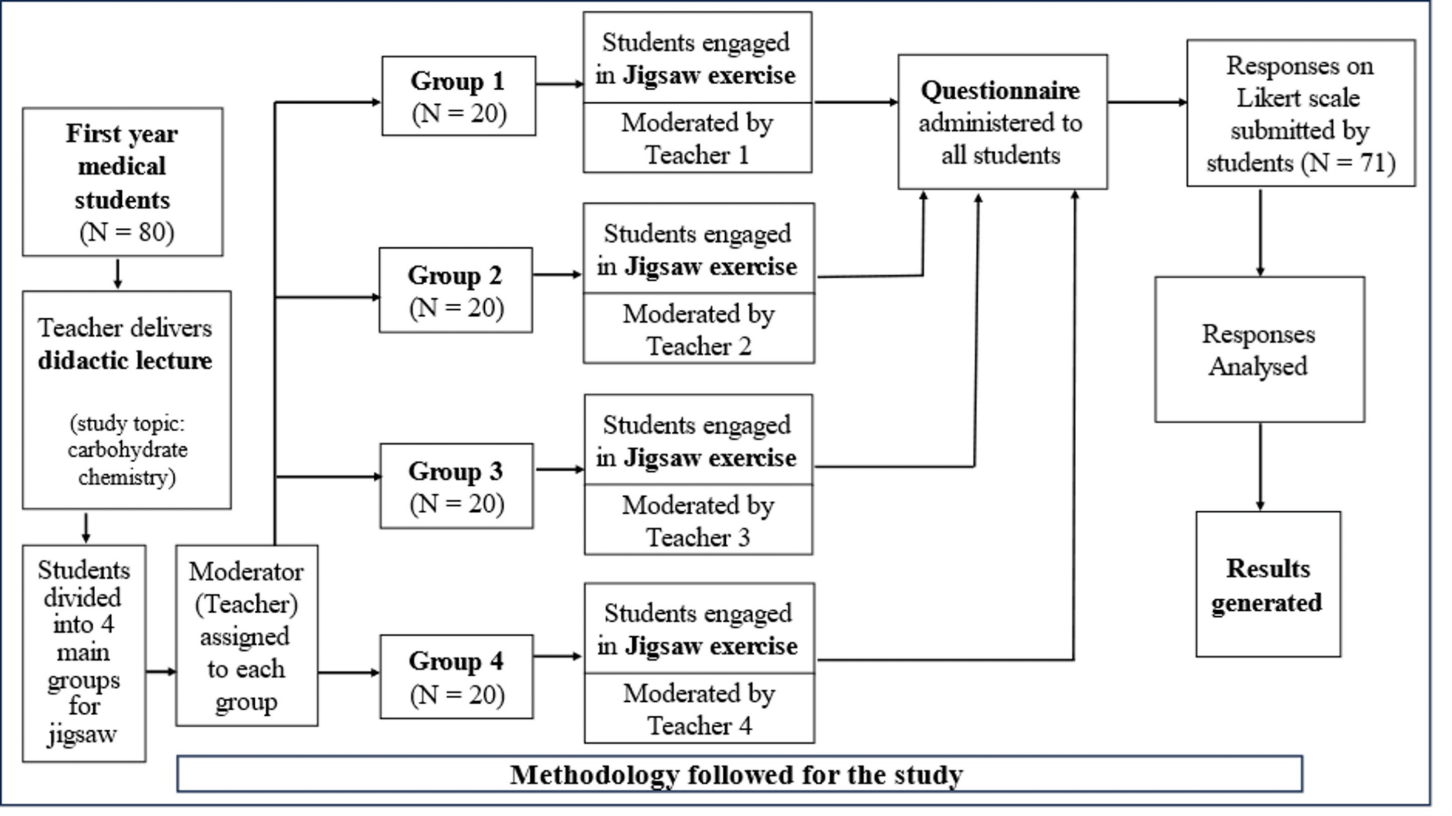
Steps showing the methodology used for carrying out the study

**Figure 2:**
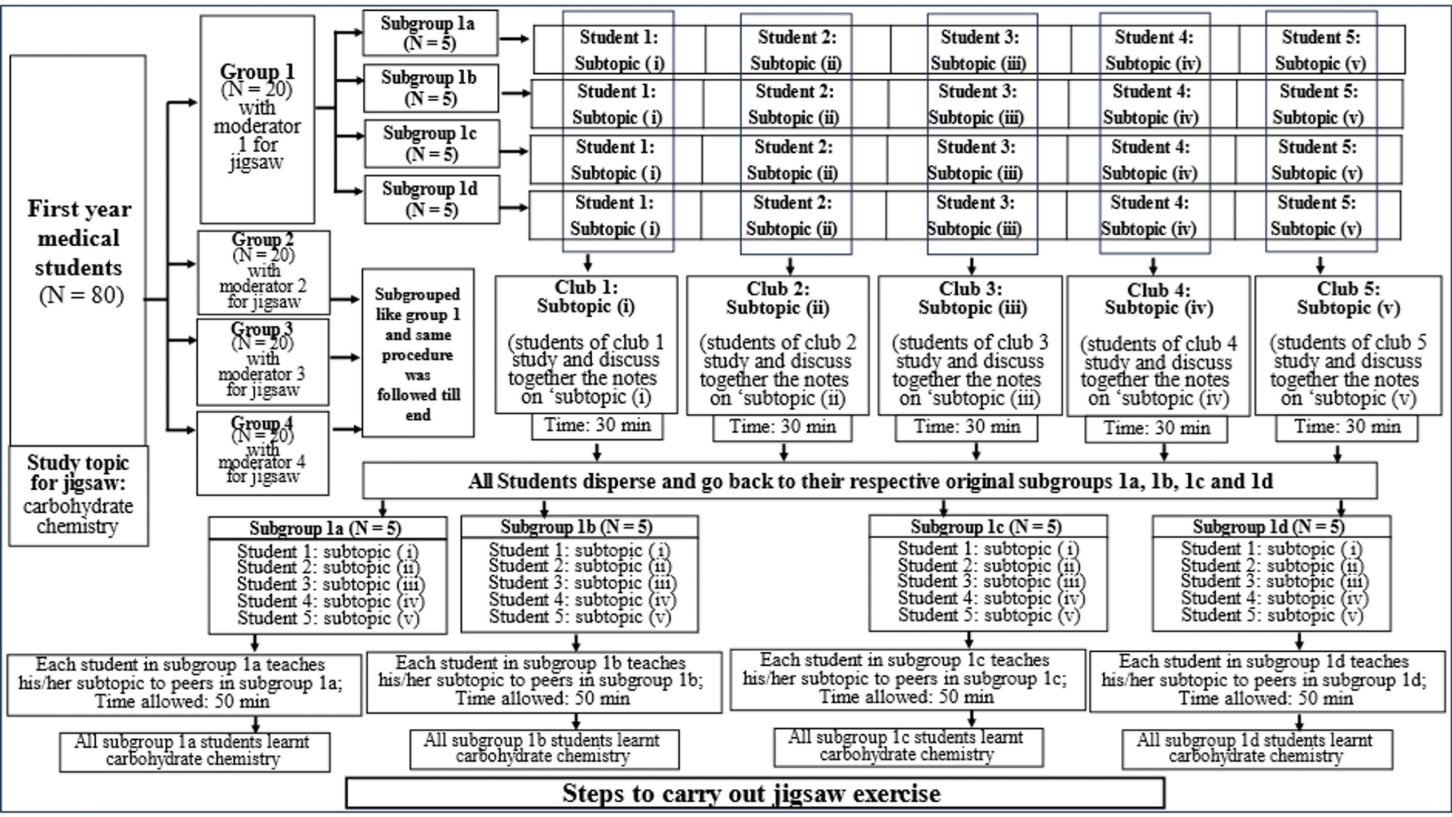
Steps showing how the jigsaw exercise was conducted on first-year medical students

The study was approved by the Institutional Ethics Committee of All India Institute of Medical Sciences, Bathinda (approval number: IEC/AIIMS/BTI/306, approval date: March 3, 2023). The study was conducted in our medical college in the Department of Biochemistry after obtaining consent from study participants on March 9, 2023, and was completed by June 15, 2024. The study design was a cross-sectional survey study. The study participants (N=80) were first-year professional medical students. For the study, the students were initially taught a topic from their biochemistry syllabus called "carbohydrate chemistry" through the didactic lecture method in the lecture hall. The following week, the students were called for the jigsaw exercise on "carbohydrate chemistry." The methodology for carrying out the study is pictorially represented in Figure 1. The students were divided into four main groups, namely Group 1, Group 2, Group 3, and Group 4, having 20 students in each group. A teacher (faculty member) from the Department of Biochemistry was assigned to each of the four groups as the moderator for the jigsaw exercise. The activity was carried out in four separate classrooms. The steps to conduct the jigsaw exercise have been diagrammatically shown in Figure 2. They are being described here for group 1, but it was similarly carried out for the other three groups as well by their moderators. The Group 1 students (N = 20) were divided into subgroups 1a, 1b, 1c, and 1d, with five students each. The teacher then split the topic of "carbohydrate chemistry" into five subtopics (i-v), and each subgroup student was assigned one of these five subtopics. The teacher then provided the study text material (notes) corresponding to the subtopics to each student in each subgroup (Figure 2).

The students who received the same subtopics from all four subgroups were clubbed together for self-study from the provided notes and for discussion with their peers in that club to solve their doubts amongst each other. Thus, five clubs were formed when students with common subtopics were regrouped. Each club (having four students) was given 30 minutes for self-study and discussion. The moderator then asked the students to disperse and go back to their original subgroups 1a, 1b, 1c, and 1d. The moderator then instructed the students in the subgroups to teach their respective subtopics to their peers, allowing 10 minutes per student for teaching. It took about 50 minutes for all subtopics to be learned by the subgroup members, eventually holistically covering the topic of carbohydrate chemistry. Following the jigsaw exercise, the teacher distributed a self-designed peer-validated feedback form to the students to record their opinions about the jigsaw exercise. The feedback form comprised questions regarding students' perceptions and feedback about the jigsaw exercise. The students marked their responses on a five-point Likert scale in the feedback form ranging from options "strongly agree," "agree," "neutral," "disagree," and "strongly disagree."

Data analysis

In this study, 80 students participated in the jigsaw activity, but 71 students submitted completed feedback forms, representing a response rate of approximately 88.75%. The collected data was first entered into the Excel software spreadsheet (Microsoft Corporation, Redmond, WA, USA) for the organization. The feedback responses were then categorized based on the five-point Likert scale (ranging from "strongly agree" to "strongly disagree") to assess various aspects of the jigsaw exercise, such as student understanding, doubt clearing, retention potential, communication skills, and its utility as a revision tool.

The data was then analyzed using descriptive statistics. Frequencies (the number of times each response option was chosen) and percentages (the proportion of students who selected each option) were calculated for each question of the feedback form. For example, if the item in the feedback form assessed "Did the jigsaw exercise help in better understanding of the topic?" the number of students who selected each Likert scale option ("strongly agree," "agree," "neutral," "disagree," "strongly disagree") was counted to denote the frequency. Then, percentages were calculated relative to the total number of respondents. For a clearer understanding of the data, options "strongly agree" and "agree" were clubbed together. Likewise, "disagree" and "strongly disagree" were clubbed together.

## Results

It was found that 71 students out of 80 study participants filled out the feedback form, which was calculated as the response rate of the study survey. In our study, the response rate was found to be 88.75%, which was very high.

Table 1 shows the results of students' perceptions regarding the effectiveness of the jigsaw exercise. Table 2 shows students' feedback regarding implementing the jigsaw exercise in medical education.

**Table 1:** Students’ perception regarding the effectiveness of the jigsaw exercise (N=71)

	Disagree or strongly disagree (frequency (N) and percentage (%))	Neutral (frequency (N) and percentage (%))	Agree and strongly agree (frequency (N) and percentage (%))
Did the jigsaw exercise help you better understand the topic?	0 (0%)	12 (16.9%)	59 (83.1%)
Did the jigsaw exercise help you clear your doubts and concepts while discussing them with peers during the activity?	2 (2.8%)	15 (21.1%)	54 (76.1%)
Did the jigsaw exercise improve your retention potential of the topic?	4 (5.6%)	12 (16.9%)	55 (77.5%)
Did the jigsaw exercise improve your communication skills?	4 (5.6%)	8 (11.3%)	59 (83.1%)
Is jigsaw a good method for helping you revise the topic?	3 (4.2%)	10 (14.1%)	58 (81.7%)

**Table 2:** Students feedback regarding the implementation of the jigsaw exercise in medical education (N=71)

	Disagree or strongly disagree (frequency (N) and percentage (%))	Neutral (frequency (N) and percentage (%))	Agree and strongly agree (frequency (N) and percentage (%))
Should the jigsaw activity be included in your medical curriculum?	3 (4.2%)	12 (16.9%)	56 (78.9%)
Should the jigsaw activity be conducted more frequently?	8 (11.3%)	14 (19.7%)	49 (69.0%)

The results of this study (Table 1) show that the majority of the students (83.1%; 59 out of 71 students) have reported that they strongly agreed or agreed that the Jigsaw exercise helped in better understanding of the lecture topic, whereas 16.9% of the students (12 out of 71 students) gave a neutral response. There were no students who disagreed or strongly disagreed about this.

It was also found that 76.1% of the students (54 out of 71) strongly agreed or agreed that the jigsaw exercise helped them clear their doubts and concepts while discussing the topics with their peers during the activity. Around 21.1% of the students (15 out of 71 students) had a neutral response, and barely 2.8% of students disagreed or strongly disagreed with this. In the feedback, the students were also asked whether the jigsaw exercise improved their retention potential. The results revealed that the majority (77.5%; 55 out of 71) of students strongly agreed or agreed, whereas 16.9% of the students (12 out of 71 students) were neutral, and just 5.6% (4 out of 71 students) disagreed or strongly disagreed.

Besides this, the data analysis showed that most of the students, i.e., 83.1% (59 out of 71 students), strongly agreed or agreed that the jigsaw exercise improved their communication skills. In comparison, 11.3% of the students (8 out of 71) responded neutrally, whereas only 5.6% (4 out of 71) disagreed or strongly disagreed.

The student's perception of whether jigsaw is a good method for helping them revise the topic was found to be that the majority of the students, i.e., 81.7% (58 out of 71 students), strongly agreed or agreed, whereas 14.1% (10 out of 71) students showed a neutral response. Barely 4.2% of students (3 out of 71) disagreed or strongly disagreed.

Besides this, the majority of students (78.9%; 56 out of 71) strongly agreed or agreed that jigsaw activity should be included in their medical curriculum, whereas 16.9% (12 out of 71) of students had a neutral response, and just 4.2% of students disagreed or strongly disagreed (Table 2).

Based on the jigsaw activity experience, most students opined that jigsaw should be conducted more frequently. About 69% (49 out of 71) strongly agreed or agreed, while 19.7% (14 out of 71) students gave neutral responses, and about 11.3% (8 out of 71) students disagreed or strongly disagreed (Table 2).

## Discussion

After carrying out the jigsaw activity on the first-year medical students in biochemistry, the student's perception regarding the effectiveness of the jigsaw as a learning method was assessed using a feedback form, which was analyzed on a five-point Likert scale. The response rate of the study survey of the students was very high (88.75%), reflecting students' enthusiasm for the study. The study's results clearly showed that most students found jigsaw exercises useful. Invariably, for all questions asked of the students regarding the usefulness of the exercise, it was observed that more than 75% of the students either strongly agreed or agreed, making it evident that jigsaw helped in better learning. The possible reason why the jigsaw exercise helped the majority of students (59 out of 71, 83.1%) in better understanding of the topic (Table 1) could be because it is a self-directed learning exercise that engaged the students in putting active self-effort into understanding the topic, evoking their higher mental functions like critical thinking and reasoning, and subsequently teaching the topic to the peers, thereby reinforcing the recall process. Other studies [8,9] also reported that self-directed learning is better than passive learning. Dnyanesh et al., in their study, also confirmed that the students found the jigsaw session to be more engaging than traditional classes, and the scores were higher in the jigsaw method study group than in the control group [10]. In their study, Fissler et al. tested the jigsaw method and found that puzzling stimulates multiple cognitive abilities and protects against cognitive aging [11].

Besides this, most students (54 out of 71, 76.1%) in our study opined that the jigsaw exercise helped them clear their doubts and concepts through peer discussion (Table 1). This finding contrasts the usually observed trend in the traditional didactic lecture method, a one-way mode of communication wherein the students hardly get any individual attention from the teacher as a chance to clear their doubts [12]. Moreover, the didactic lecture method, being a monologue, lacks active interaction between the students and the teacher, eventually hindering the development of communication skills in students during the learning process [13]. However, in our study, 83.1% of students (59 out of 71; Table 1) opined that the jigsaw exercise improved their communication skills too. Additionally, a recent study by Nazari et al. highlighted that the jigsaw method is a peer-to-peer teaching method that offers students a chance to be teachers for their peers and effectively improves learning among medical students by building their communication skills and better prepares medical students for their careers as future clinicians [14]. A study by Buffalari et al. has also reported that active learning leads to the development of communication skills [15], a much-needed essential skill in a doctor for establishing a good doctor-patient relationship in the medical profession [16]. Chopra et al. also found results in conformity with ours, stating that jigsaw activity was found to be helpful for 70% of students to overcome shyness and hesitation in class, thereby improving their communication and teaching skills [17].

Moreover, it is imperative that the teaching-learning method that the teacher chooses to teach a topic should enable the learners to retain the information for a longer time, especially because the syllabus of the medical course is vast, and the quality of medical education is yet maintained. In our study, 77.5% of the students (55 out of 71) opined that the jigsaw exercise improved their retention potential (Table 1). Williams et al. tested a jigsaw activity in their study that was aimed at minimizing memorization and increasing knowledge retention by creating interest in biochemistry topics through the jigsaw activity and found that to be effective in their study [18]. This can be attributable to the activation of the cognitive processes and active engagement as a team. Biochemistry is a subject taught to medical undergraduates in the first professional year of their medical program, wherein the students often find an immediate shift in their education pattern compared to school. The subjects in a medical course are more of practical importance rather than theoretical, and the mode of assessment is also usually a combination of various types of questions, such as subjective essay-type theory questions, viva voce, OSPE, MCQs, case study-based questions, spots, quizzes, assignments, etc. Thus, the method deployed to teach the students should be such that the students should retain the information longer, and it should be noted that not all students have the same intellectual capacity to adjust to this paradigm shift and effectively imbibe the information taught. Biochemistry is a subject that has detailed pathways and reactions for the students to memorize, and the clinical exposure of students to the patients is quite limited, which makes the subject additionally boring; therefore, a jigsaw exercise can be a good exercise for students to learn the reactions, pathways, and concepts through peer discussion in an effective manner. In our previous study, we found that the jigsaw exercise effectively increased the knowledge and retention of the students, especially those not high achievers academically [19]. Other studies have also found the jigsaw method to be an effective learning exercise, the results of which are in accordance with ours [20,21].

Besides this, most of the students (58 out of 71; 81.7%) in our study (Table 1) opined that jigsaw can be a good revision exercise. The reason for this finding could be that the didactic lecture, in the first instance, already acquainted the students with the topic superficially, which might or might not have created sufficient interest in the students for further self-study owing to its monotonous nature [22]. However, when the jigsaw activity was planned after the lectures, it provoked active learning in students through discussions and teamwork [23] through group activity, leading to a recapitulation of the information imparted through lecturing, besides building a deeper understanding of concepts through self-directed learning. Mohebbi et al. also found that blended teaching through flipped classrooms and jigsaw was more effective than lecturing alone [24]. Other researchers have reported that self-directed learning exercises facilitate lifelong learning by building team-building capacity, communication skills, conflict management approaches, leadership skills, and time management by receiving inputs from peers and facilitators after self-directed learning [9,25].

The analysis of the responses (Table 2) submitted by the students also showed that the majority of students (56 out of 71; 78.9%) wanted jigsaw activity to be included in the medical curriculum based on their present study experience. Furthermore, research by Bucklin et al. showed that more efforts are needed to increase innovation and incorporate evidence-based active learning strategies in medical education, especially to foster learner engagement, critical thinking, and problem-solving ability [26]. Besides this, most of the students in our study (49 out of 71; 69.0%) also opined that jigsaw exercises should be conducted more frequently. Thus, considering the results of other researchers who have tested the jigsaw technique in their respective domains [27-29], the jigsaw method seems promising to serve as a good active teaching-learning strategy in training medical students.

Limitations of study

The jigsaw exercise is a small-group activity. Since the number of participating students was large, multiple moderators and classrooms were required to conduct the activity in multiple small groups, which could be a major limitation in conducting the study. Moreover, the session required two teaching hours to conduct the jigsaw activity, besides the substantial amount of time spent pre-planning the study and printing the handout notes. In our study, a jigsaw exercise was implemented on just one topic, "carbohydrate chemistry," due to time limitations and moderators' availability. However, the authors suggest that future studies should explore using the jigsaw method for other complex topics, such as metabolism or enzymes. Additionally, it was observed that some students struggled to teach their peers, particularly those who were less fluent in English. Nevertheless, the authors believe regular engagement in such activities would help these students gradually improve their communication skills.

## Conclusions

The jigsaw exercise was found to be a useful learning exercise by the first-year medical students, as evidenced by their responses, which helped them better understand and retain the topic. Besides that, jigsaw may be used as a group activity to revise the topic and enhance self-directed learning among students. The authors recommend introducing jigsaw exercises into the medical curriculum based on the students' feedback and potential advantages.

## Appendices

Annexure: feedback form

Choose from the given options to mark your response:

**Table 3:** Feedback form for assessing students' opinion of the jigsaw activity

S. No.	Questions	1. Strongly disagree	2. Disagree	3. Neutral	4. Agree	5. Strongly agree
1	Did the jigsaw exercise help me better understand the topic?					
2	Did the jigsaw exercise help you clear your doubts and concepts while discussing with peers during the activity?					
3	Did the jigsaw exercise improve your retention potential of the topic?					
4	Did the jigsaw exercise improve your communication skills?					
5	Is jigsaw a good method for helping you revise the topic?					
6	Should the jigsaw activity be included in your medical curriculum?					
7	Should the jigsaw activity be conducted more frequently?					
